# Evaluation of candidate reference genes for quantitative real-time PCR normalization in blood from red deer developing antlers

**DOI:** 10.1038/s41598-022-20676-9

**Published:** 2022-09-28

**Authors:** Camilla Broggini, Nieves Abril, Juan Carranza, Alberto Membrillo

**Affiliations:** 1 Wildlife Research Unit (UIRCP-UCO), University of Cordoba, 14014 Cordoba, Spain; 2 Department of Biochemistry and Molecular Biology, University of Cordoba, Cordoba, Spain

**Keywords:** Biochemistry, Molecular biology

## Abstract

Sexual selection favors male traits that increase their ability to monopolize the breeding access to several females. Deer antlers are cranial appendages that regenerate annually in males. Throughout life, the phenology of antler growth advances and antler mass increases until the stag reaches, between 8 and 10 years old, maximum body mass and highest reproductive success. The molecular mechanisms of antler development are of great interest in both evolutionary and regenerative medicine studies. To minimize errors in the assessment of gene expression levels by qRT-PCR, we analyzed the stability of a panel of eight candidate reference genes and concluded that qRT-PCR normalization to three stable genes is strongly convenient in experiments performed in red deer antler blood. To validate our proposal, we compared the expression level of three genes linked to red deer antler growth (ANXA2, APOD and TPM1) in fifteen male red deer classified as young (up to 4 years old) and adults (4–6 years old). Our data confirms that B2M, ACTB and RPLP0 are valuable reference genes for future gene expression studies in red deer antler blood, which would provide increased insight into the effects of intrinsic factors that determine antler development in red deer.

## Introduction

Males of polygynous species often develop weapons and ornaments to confront rivals and attract potential mates^1^. The antlers of male red deer (*Cervus elaphus*) are an example of sexually selected appendages that undergo annual regeneration and are associated with reproductive success^2^. Antlers are usually shed in early spring, and they immediately start to regrow with an exceptionally fast development from late spring until August, when they stop growing and ossify before males can use them to fight against rivals during the rutting season^3,4^. The extraordinary characteristics of antler rapid growth and annual regeneration make red deer a valuable model not only for studies in ecology, behavior, evolution and biology, but also for the study of mammalian tissue regeneration^5,6^.

Antler size is notably heritable (e.g. 0.33 in Rum red deer^2^) and antler morphology has been shown to depend on pleiotropic effects of multiple genes that maintain variation within populations^7^. However, antler development also depends on age and environmental factors like nutrition^8^, and it has been demonstrated that antler size is also modulated according to the level of mating competition in the social environment^9^.

In the last decades, some growth factors and cell signaling pathways controlling antler regrowth have been well established in deer and scientific advances have driven the identification of several antler-specific genes^10–13^ and proteins^14–16^ that are involved in this rapid growth.

Gene expression analysis quantifies the formation of a gene product from its coding gene. The development of high-throughput technologies has allowed the large-scale quantitative analysis of gene expression changes related to the regulation of antler growth.

Along the last 20 years, heterologous microarray hybridization^17^, amplified fragment length polymorphism^18^, DNA chip microarray^19^, Affymetrix human U133plus 2 Genechip^20^ or RNA-Seq^21^, have been applied to discover the mechanisms regulating antler growth. However, these studies mainly used Chinese sika deer as the model organism, focusing on the study of the different antler tissues and often generating some conflicting results^22^ thus calling for further studies in red deer to provide higher quality evidence from well-designed and executed analyses to decipher the mechanisms and factors regulating the growth of antlers, a sexual trait critical for breeding success.

To our knowledge, to date no one has used quantitative real-time PCR (qRT-PCR) to correlate blood transcriptional profiles with antler growth in red deer. Furthermore, the choice and selection of suitable reference genes used for standardization is critical and they must be validated for reliable quantification.

Sensitivity, reproducibility, wide dynamic range, specificity, and accuracy have made fluorescence-based qRT-PCR the gold standard for quantifying the amount of mRNA transcripts in biological samples during gene expression analysis. Numerous factors (RNA purity and integrity, genomic DNA contamination, pipetting errors, primer concentration and efficiency) can affect qRT-PCR results. Therefore, the generation of accurate, reproducible and precise results requires that qRT-PCR experiments are carried out after a rigorous analysis of the stability of the optimal genes to be used as references for data normalization and validation of the chosen reference gene/s under given experimental conditions^23^. Improper reference genes used in data processing may lead to inaccurate and even wrong results^24^.

However, a limited number of studies can be found in the literature that evaluated the stability of expression of various genes using more than two approaches in red deer and none in the antler blood. In recent years, increasing evidence has shown that two or more reference genes need to be evaluated for stable expression for use in normalizing expression data^25^. In an experimental design with multiple samples, a balance needs to be reached between the use of multiple genes in multiple samples and the degree of accuracy required^26^. Here we analyzed the expression stability of eight commonly used reference genes to identify those most suitable for qRT-PCR normalization in experiments performed on red deer antler blood collected when antlers are cut, using a comprehensive statistical approach (RefFinder). To validate the selected reference genes, the expression level of three genes related to red deer antler growth (ANXA2, APOD and TPM1) was compared in fifteen red deer males classified into two groups according to their age, with one group (control) composed of young males (up to 4 years old) and the other one of adult males (4–6 years old). The rationality behind this comparison was the known effect of age over annual antler growth: antler growth phenology advances and antler mass increases with age until around 8–10 years of age^3,9^, the average age of a male red deer picking up in body mass and reproductive success.

The aim of this work was therefore to find valuable reference genes for future gene expression studies in red deer blood from antlers in the last stage of growing and advanced mineralization, which would provide insight into the molecular mechanisms of antler development.

## Materials and Methods

### Red deer specimens and sampling sites

Male red deer (*Cervus e. hispanicus*) specimens came from three different areas in Spain: Lagunes deer farm and Cabañeros National Park (Dehesa del Carrizal), both in Ciudad Real province (Castilla La Mancha region), and El Pardo in the Community of Madrid (Supplementary Table S1). None of the animals showed clinical signs of disease. Data and blood samples were collected in 2019 and 2020 during antlers cutting, a legal traditional management activity usually conducted in July (when antlers have almost stopped growing but still have blood supply) to protect other animals and handlers from injury. Since a deer > 4 years old is considered mature, we divided our samples into two categories: young (n = 5; up to 4 years old) and adult (n = 10, 4 years and older).

Our access to these farms was approved by their management authorities. No deer were culled or were his antlers cut for the purpose of this study. Antler cut was carried out at the end of growing and mineralization, under the supervision of a veterinary. Ethical issues were revised and approved by the Wildlife Research Unit (UIRCP-UCO), University of Cordoba. All our methods were performed in accordance with relevant guidelines and regulations. In addition, the study was carried out in accordance with ARRIVE guidelines.

### Antler blood samples

Blood samples were collected from the base of the antler at the antler cut-off time (dehorned blood) in 10 ml EDTA-containing tubes and immediately transferred to RNAlater solution (500 µL blood per 1.3 mL of preserver) for RNA stabilization.

Samples were kept at 4 °C for less than 3 days and then stored at − 20 °C till use.

### RNA extraction, quantification, quality measures and cDNA synthesis

Total RNA extraction from dehorned blood samples preserved in RNAlater was carried out using the commercial RiboPure-Blood Kit (Thermo Fisher Scientific) according to the manufacturer’s protocol. Briefly, samples (500 µL blood per 1.3 mL of preserver) were centrifuged (1 min, 16,000 *xg*) and the supernatant was carefully and thoroughly removed. Sample pellets were lysed with (800 μL) Lysis Solution and (50 μL) Sodium Acetate Solution and vigorously vortexed. Then, 500 μL of Acid-Phenol:Chloroform were added, samples were vortexed for 30 s and the mixture stored at room temperature for 5 min and centrifuged (1 min, 16,000 *xg*). The aqueous (upper) phase containing the RNA was transferred to a new tube, thoroughly mixed with 600 μL (~ one-half volume) of 100% ethanol, applied in successive rounds of ~ 700 μL to a Filter Cartridge assembly and centrifuged (5–10 s, 16,000 *xg*). The RNA retained in the column was washed, first with 700 μL of Wash Solution 1 and then, twice with 700 μL of Wash Solution 2/3. Finally, the Filter Cartridge was transferred to a new tube and the RNA eluted with (50 μL) Elution Solution (preheated to ~ 75 °C), denatured by heating at 55–60 °C for 10 min followed by rapid cooling on ice for at least 5 min, and kept at -80 °C till use. The concentration and purity of RNA preparations were determined spectrophotometrically. An Agilent 2100 Bioanalyzer (Agilent Scientific Instruments) was used to determine the integrity of the isolated RNA and assign an RNA Integrity Number (RIN) to each sample.

gDNA removal and cDNA synthesis were achieved by using the QuantiTect Reverse Transcription Kit (Qiagen), following the manufacturer’s instructions. The cDNA was diluted to 25 ng/uL (qRT-PCR working solution) and stored at − 80 °C until use.

### RT-qPCR

This study was carried out to conform to the Minimum Information for Publication of Quantitative Real-Time PCR Experiments^27^.

#### Selection of reference genes

Putative reference genes were selected based on information about their common use as references in the literature (Supplementary Table S2).

Primers were designed using red deer gene sequences deposited in the GenBank database (NCBI, https://www.ncbi.nlm.nih.gov/genbank/) with OLIGO 7 Primer Analysis Software (Molecular Biology Insights, Inc., http://www.oligo.net) as previously described^28^. In addition to being free of hairpins and duplex structures, primers were required to have a high Tm to ensure specificity^29^. The sequences and some characteristics of the primer pairs used for specific amplification of selected genes, as well as the PCR conditions used for transcript quantification are listed in Table 1. Table 1 also includes data about the primers specific for amplification of the genes ANXA2, APOD and TPM1, used in this work for validation of the selected reference genes. Before being used in qRT-PCR, primers specificity was evaluated by 1% agarose gel electrophoresis of their PCR amplicons, which were also sequenced and then compared with those in the GenBank database.

**Table 1:** Primer pairs used in this work.

Gene symbol	Accession number	Primer sequences (5′ → 3′)	Junction	Amplicon length (bp)	E^2^ (R^2^)	Ref
**Reference genes**			Exon			
SDHA	OWK02252.1	F: CGCTCAAGGAGCCCTGCCCTGCAC	3	118	98.7 (98.3)	This work
		R: GGCATGCCGTAATTCTCCAGGCGTCCCC				
PGK1	OWJ99526.1	F: GGGGAAGCGGGTCGTCATGAGGATCAAGGC	3	117	97.6 (98.8)	This work
		R: GGGACACCATCAGGCCGGCCCAGG				
GAPDH	OWK03708.1	F: ACTGCTTGGCCCCCCTGGCCAAGGTC	6	147	99.6 (99.1)	This work
		R: GGGCAGCCCCTCGGCCATCACGCCAC				
ACTB	DQ233465.1	F: GATCTGGCACCACACCTTCTA	3	217	96.5 (97.0)	^18^
		R: CCCAGAGTCCATGACAATACC				
RPLP0	OWK14870.1	F: GCCTCACATCCGGGGGAACGTGGGCTT	5	155	101.4 (99.3)	This work
		R: CCAGACCGGTGTTCTGGGCCGGCACAGTGA				
GUSB	OWK11092.1	F: CTTCTCCGACAACCGGCGCCAGGGCT	1	122	100.1 (100)	This work
		R: GCCCGTCCTGGCCCACGTCGTTGAAACTTG				
B2M	OWK09923.1	F: CCTGCTGTCCCACGCTGAGTTCACCC	2	77	98.7 (100)	This work
		R: CTGCGAAAGTAATGTGCTTCACTCGGCAGC				
G6PD	XM_043895505.1	F: GGACCTCCCCGGGGCCCCGCGCCGAAAG	1	102	96.5 (97.3)	This work
		R: CGCGAGTCCGCCTGCAGCTCCCCGCAGCTC				
**Bone growth related genes**						
APOD	XM_043874311.1	F: CGCCCTCGTGTACTCCTGTACCACGAT	5	219	98.2 (99.5)	This work
		R: ATGGAGTGAATGCAGCTCCCTTTAGAGCCT				
TPM1	DQ239919.1	F: GCCATTTCCCAAATTGACAT	14	211	95.3 (97.3)	^18^
		R: CCACAGTGGGACCTTTTGTT				
ANXA2	DQ239920.1	F: CTTCCGCAAGCTGATGGTCGCCCTCGC	7	167	97.5 (99.6)	This work
		R: CGCTCCGCTCGGTCATGATGCTGATCCAC				

Transcript quantification by real-time qRT-PCR was performed according to the recommendations of the Minimum Information for Publication of Quantitative Real-Time PCR Experiments (MIQE, https://www.gene-quantification.de/miqe-index.html)^27^ as previously described^30^. The PCR protocol was adapted to the different Tm of each primer pair.

Genes Phosphoglycerate Kinase 1 (PGK1), Glyceraldehyde-3-Phosphate Dehydrogenase (GAPDH), Ribosomal Protein Lateral Stalk Subunit P0 (RPLP0) and Glucuronidase Beta (GUSB) were amplified using a two-step protocol composed by a 98 °C, 4 min polymerase activation step and 40 cycles including a denaturation (95 °C, 15 s) step and a hybridization/extension (70 °C, 30 s) step.

A hybridization/extension (65 °C, 30 s) step was used for Succinate Dehydrogenase (SDH), Beta-2-microglobulin (B2M), Glucose-6-Phosphate 1-Dehydrogenase (G6PD), apolipoprotein D (APOD) and annexin 2 (ANXA2) genes. Finally, genes ß-Actin (ACTB) and α-tropomyosin (TPM1) were quantified with a classical three step protocols: denaturation (95 °C, 15 s), primer annealing (60 °C, 30 s) and target elongation (70 °C, 15 s). All samples were quantified in quadruplicate in a CFX-96 Touch Real-Time PCR Detection System (Bio-Rad), by using 50 ng of cDNA per reaction (20 μL) and the SsoAdvanced Universal SYBR Green Supermix (Bio-Rad), following the manufacturer’s instructions. A melting curve analysis from 65 to 95 °C was applied to all PCR reactions to ensure specificity of amplification in each PCR reaction.

Before reading the C_T_ values, the absence of signal in the negative control (non-template sample) was tested. When this was the case, the C_T_ values generated by qRT-PCR were transferred to Microsoft Excel and the mean of the quantification cycles was calculated as the average of the four replicates if the SD was less than 0.2; otherwise, the C_T_ value of the outlying sample was removed from the study and the remaining data (at least n = 3) were reanalyzed.

The efficiency of amplification of each primer pair was evaluated by amplifying in quadruplicate a tenfold dilution series (resulting in a concentration range from 20 to 2 × 10^5^ pg) of a mixture of all cDNA samples^31^. The slope of the standard curve obtained by plotting the obtained C_T_ values against the RNA amount per well was used to calculate the efficiency according to the equation E = 10^(−1/slope)^ − 1.

### Analysis of the expression stability of candidate reference genes

The cycle threshold (C_T_) values generated from qRT-PCR for all the samples and putative reference genes (Supplementary Table S3) were transferred to Microsoft Excel and used to analyze the expression stability of candidate reference genes by using the statistical algorithm software programs geNorm^32^, NormFinder^33^, BestKeeper^34^, the comparative ∆C_T_ method^35^ and the comprehensive web-based analysis tool RefFinder^36^. C_T_ data in Microsoft Excel format were used to evaluate expression stability via the ΔC_T_ method or they were directly imputed into the BestKeeper or RefFinder software. For geNORM and NormFinder use, the C_T_ values were first used for calculation of linear relative values (keeping the lowest relative quantity for each gene as one), which were then imported into software to calculate gene expression stability value (M) and to rank genes according to their M value. The cut-off M value was set at 1.0, with a lower M value indicating more stable expression.

#### Confirmation of the suitability of selected reference genes

Three genes related to antler growth, ANXA2, APOD and TPM1 were selected as target genes to validate the reliability of identified reference genes in blood samples of young (n = 5) and adults (n = 10) specimens of males red deer. ANXA2 and APOD can be linked to the robust development of the antler while TPM1 slows down the vigorous cell proliferation making conditions favorable toward differentiation^18^.

Average C_T_ values were calculated from all the biological replicates in each experimental condition, and from three technical replicates used for relative expression analyses. Relative quantification of target genes in different samples was carried out using the comparative ∆∆C_T_ method according to the equation *Fold variation* = *(1* + *E)*^*−ΔΔC*T^ where E represent the efficiency of amplification of the primer pairs, and the most stable reference genes identified were used for normalization.

### Statistical analysis

Comparison of data between the young (control) and the adult groups was carried out with Dunnett’s test using InStat v.3.05 (GraphPad software Inc., CA, USA. https://www.graphpad.com). All results were evaluated using an unpaired Student’s t-test, and differences with p < 0.05 were regarded as statistically significant.

## Results and discussion

Here we have performed a stability analysis on a selected group of putative reference genes, according to MIQE Guidelines^27,37^, to find adequate reference genes for future gene expression studies by qRT-PCR in red deer dehorned blood that can provide a better insight into the molecular mechanisms of antler development and the effects of intrinsic and environmental factors determining antler size and hence potential reproductive success of male red deer.

### Quality and integrity of RNA samples

To ensure RNA quality, blood samples were collected and preserved in RNAlater solution for a short time (less than 3 days) and then kept frozen till use. RNA was extracted with the RiboPure-Blood Kit following the manufactures recommendations.

**Figure 1:**
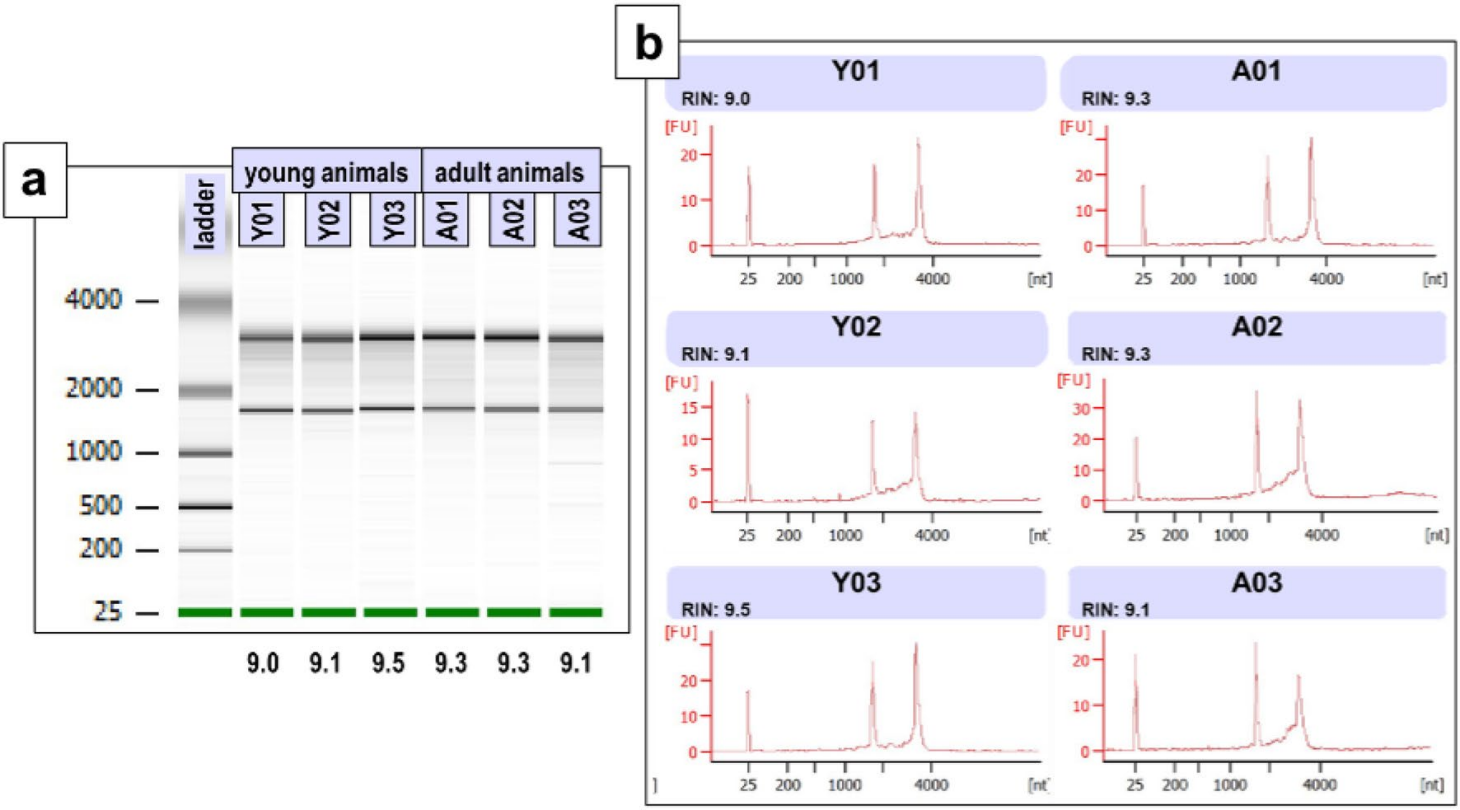
RNA integrity. Gel images (**a**) and electropherograms (**b**) of three representative blood RNA samples in each age group and the RIN values (under the green lines) obtained with the 2100 Bioanalyzer (Agilent). RIN = RNA Integrity Number. Green lines = Bioanalyzer internal marker.

RNA preparations were considered pure when their ratios of absorbance A260/A280 and A260/A230 were in the range of 2.0–2.2. RNA integrity was evaluated by using an Agilent 2100 Bioanalyzer, which performs microfluidics-based automated electrophoresis of RNA molecules and assigned the samples to 10 different categories ranging from 1 (RNA totally degraded) to 10 (RNA intact) through the RNA Integrity Number (RIN). The RIN values of all samples in this study equaled or exceeded 9.0, indicating that our RNA samples were of sufficient quality to be used in transcript quantification by qRT-PCR. A representative example of RNA integrity of our blood samples is shown in Fig. 1.

To ensure absence of contaminant gDNA, we used a cDNA synthesis kit that included a pretreatment with DNase-I, a retro-transcriptase with high RNA affinity and a RT-primer mix containing both oligo-dT primers and random hexamers.

This kit enables high yields of cDNA from any RNA template, provides great sensitivity in the detection of low-abundance genes and allows high reproducibility in real-time RT-PCR (https://www.qiagen.com/es/resources/resourcedetail?id=f0de5533-3dd1-4835-8820-1f5c088dd800&lang=en).

### Amplification specificity and efficiency

The success of the polymerase chain reaction (PCR) is highly dependent on primer design, as primers are the main determinants of its specificity, sensitivity, and robustness.

**Figure 2:**
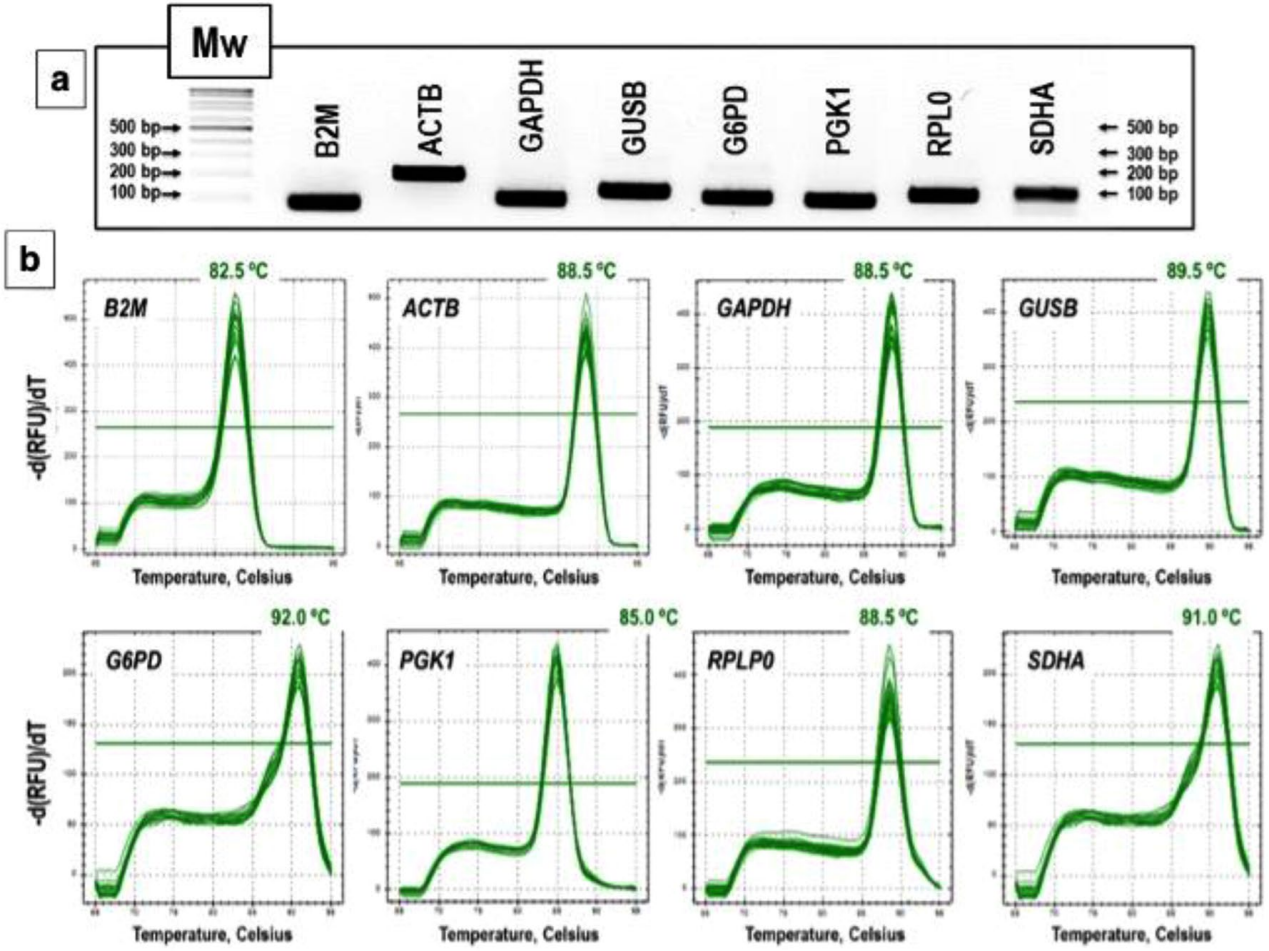
Gene specificity and amplicon size and Tm. (**a**) Agarose gel (2%) of qRT-PCR products for each gene showing one band of the expected size. Equal amounts of cDNA from each sample were mixed and amplified with each primer pair and the PCR products loaded on the gel. A standard DNA molecular weight ladder was also loaded. (**b**) Melting curves of the 8 putative reference genes showing a single peak and the Tm values of each amplification product. An uncropped version of the gel in (**a**) is depicted in Supplementary Fig. 1.

We used the Oligo 7 to design primer pairs that are specific for their target sequence and that do not form hairpins, self- or hetero dimers. We optimized PCR conditions for each primer pair (annealing temperature and concentration) to achieve the best amplification. Primer’s specificity was then experimentally evaluated by nucleotide sequencing of the amplicons, by visualizing the PCR products in agarose gels and by carrying out melting curves at the end of the PCR reaction. All primer pairs listed in Table 1 produced a specific amplicon of the expected sequence and size and exhibited a single sharp peak in the melting curve (Fig. 2).

The amplification efficiency (E) of PCR is defined as the fraction of target molecules that are copied in each PCR cycle, with E = 1 (or 100%) meaning a perfect duplication of the number of template DNA molecules in each PCR step. However, there are many factors that can influence PCR efficiency, such as the primers used or the presence in the sample of inhibitors derived from the regents used in the retro-transcription step. Hence, a PCR efficiency analysis is mandatory.

The estimation of PCR efficiency was achieved by means of a standard curve constructed by a tenfold serial dilution (concentration range from 20 to 2 × 105 pg total cDNA per reaction) of a mixture of all cDNA samples. A plot of the C_T_ values versus the logarithm of the target concentrations was used to calculate the efficiency of amplification of each primer pair. The amplification efficiencies of all the primers ranged from 95.3 to 101.4%, with R2 ranging between 0.9701 and 1.0000 (Table 1).

### Stability analysis of putative reference genes

qRT-PCR data are normalized using reference genes to correct for differences in the amounts of template cDNA. Appropriate reference gene(s) selection is a critical and challenging issue since incorrect reference gene selection can distort results leading to false interpretations. To be used as a reference gene, a gene must show no expression changes between the samples to be compared and therefore reference genes must be carefully selected based on the experimental data. Several bioinformatics tools are commonly used to find the most stable reference genes and determine the minimal number of reference genes to be used in each qRT-PCR experiment.

Very few studies can be found in the literature that assessed the expression stability of various genes in red deer and even less in red deer antler blood. In the works by Harrington^38,39^ the suitability of B2M was established by accepting an average fold-change of less than two between C_T_ values of samples. Another study^40^ only used two candidate genes with just two methods (geNorm and NormFinder) to identify one of our selected reference genes (B2M) as suitable for accurate and reproducible qRT-PCR analysis of gene expression in red deer blood. None of the statistical algorithms used to date to assess the stability of gene expression cover all the variables associated with gene expression studies; therefore, drawing conclusions based on one or two methods can lead to false positives and incorrect conclusions^41^. To avoid that, we used here a comprehensive statistical approach (RefFinder) to determine good reference candidates for reliable normalization of gene expression data in red deer antler blood. Following the protocol in the MIQE guidelines^27,37^, we determined the C_T_ values for each candidate gene in the fifteen mRNA samples from antler blood of both young and adult red deer specimens. The obtained C_T_ values, *i.e*., the number of cycles required for fluorescence to reach the fixed threshold level, for all samples and genes are listed in Supplementary Table S3.

Expressions levels of the putative reference genes showed B2M being the most abundant (lowest C_T_ value) and G6PD the least abundant. Descriptive statistics were performed with Excel complement XLSTAT v. 2020.2.2 software (Addinsoft).

**Figure 3:**
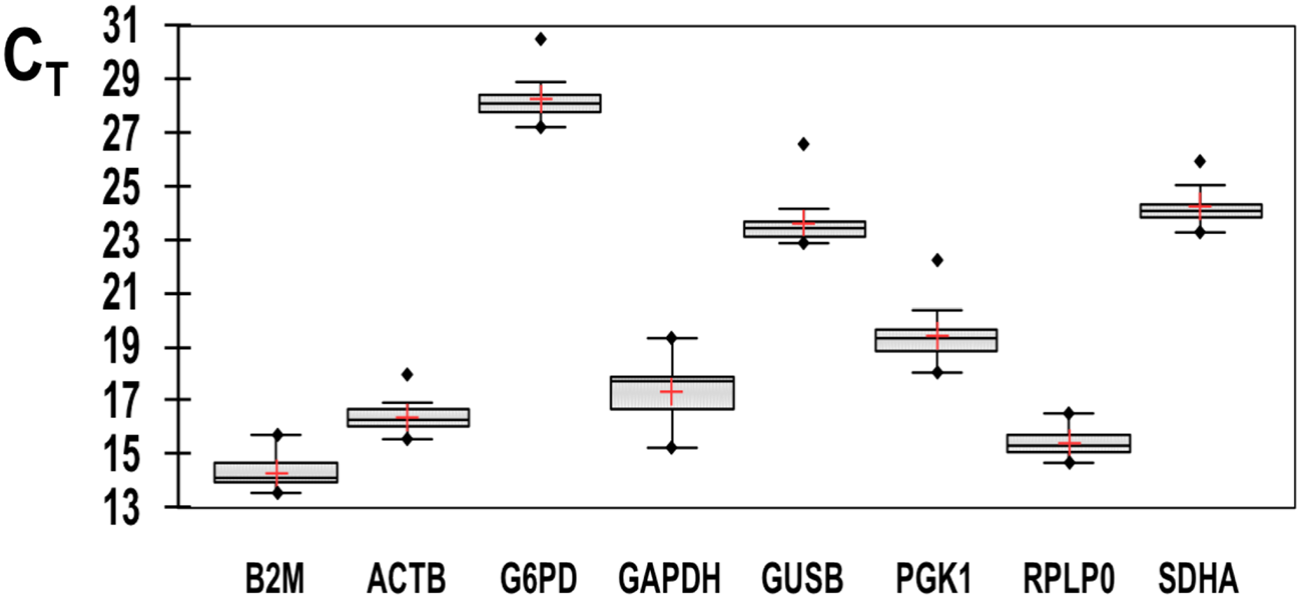
Distribution of CT values for candidate reference genes in red deer blood samples. Boxes: range of CT values; black center line: median CT; cross: mean score; upper and lower hinges: 75 and 25 percentiles; whiskers: largest/smallest CT values within 1.5 times IQR (Interquartile range) from the upper and lower hinges.

The results are listed in Supplementary Table S4 and presented in Fig. 3.

The statistical parameters of the C_T_ values indicate that GAPDH is the gene with the most variable expression levels, as deduced from its higher standard deviation (SD). All other genes showed remarkable stability, with SDHA showing the most stable expression levels. This gene showed intermediate mean C_T_ values, making it likely to be a suitable reference gene in our study with antler blood samples of both young and adult red deer.

**Figure 4:**
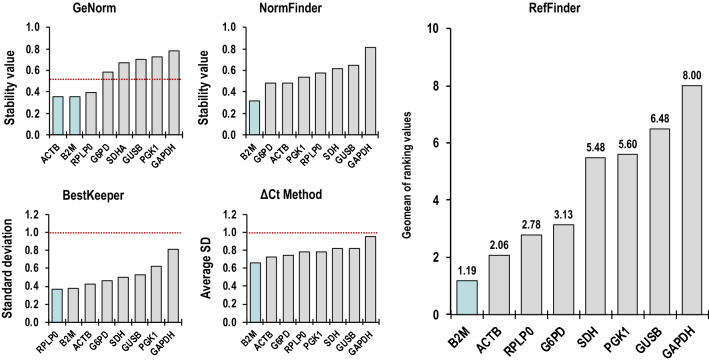
Stability ranking of candidate reference genes. The final overall ranking of the eight candidate reference genes using the RefFinder tool, and the expression stability of the genes calculated using geNorm, NormFinder, BestKeeper and the comparative ΔCT (available in RefFinder). Within each graph, the most stable gene is on the left (highlighted bar) and stability decreases towards the right. The dotted line indicates the cut-off value.

To get a better insight of the gene expression stability, a more complex analysis was performed using the web-based tool RefFinder^36^, which integrates data generated by four different algorithms: NormFinder^1^, geNorm^33^, BestKeeper^34^ and the comparative ∆C_T_ method^35^ to give an overall integrative ranking of gene expression stability, a lower position in the ranking meaning greater stability (Supplementary Table S5 and Fig. 4).

We found here that B2M was the most stable gene in our samples and experimental conditions. *Β-2-microglobulin* (*B2M*) is a component of major histocompatibility complex I and used as a normalization scalar in different studies, including those on osteogenesis^42–44^. In contrast, our data show that GAPDH was the gene with the highest instability in all the used methods and in most of the algorithms.

The lack of concordance between the different approaches is due to the parameters considered by each individual algorithm to establish the order. GeNorm^32^ determines the most stable reference genes after transforming the C_T_ values into linear relative values (keeping the lowest relative quantity for each gene as one), using the comparative ∆C_T_ method and giving a gene expression stability value (M) that is used to rank genes, with a low M value indicating more stable expression: M values below 1.0 are recommended by geNorm and those with good reference genes have an M < 0.5^45^. The M values of the 8 candidate reference genes in both age group were < 1.0 (Supplementary Table S5 and Fig. 4), indicating high expression stability. In the geNorm ranking, B2M and ACTB, closely followed by RPLP0, were the three most stable genes with the lowest M values, whereas GAPDH was the most unstable gene. BestKeeper^34^ uses the C_T_s to calculate the SD values and correlation coefficient (*r*) for each gene and considers genes with SD value below 1.0 and *r* value close to one as stable in gene expression. This algorithm identified RPLP0 as the highest stable gene, closely followed by B2M (Supplementary Table S5 and Fig. 4). NormFinder^33^ identified the best reference gene based on the expression variability value, which is related to the systematic error of each candidate gene. Smaller values indicate more stable gene expression. The Δ*C*_T_ algorithm compares the relative expression of pairs of genes within each sample to confidently identify useful reference genes. It uses the average SD values to rank the stability of all candidate reference genes, considering the most stable reference gene the one with the lowest SD^35^. Both NormFinder and the comparative Δ*C*_T_ identified B2M as the best reference gene (Supplementary Table S5 and Fig. 4). Finally, the web-based tool RefFinder^36^ was employed to integrate and generate a comprehensive rank list of candidate genes based in the geomean of the ranking values generated by the geNorm, Normfinder, BestKeeper and ΔC_T_ methods. The rankings by RefFinder suggested that B2M was the most stable expressed gene, followed by ACTB and RPLP0 (Supplementary Table S5 and Fig. 4), which was largely in agreement with the other algorithms used.

### Determination of the optimal number of reference genes for normalization

To meet the requirements of accurate quantification for transcription analyses of gene expression is necessary to determine the number of reference genes to be used to produce accurate and reliable normalization^23,27,46^. We used the pairwise variation value (Vn/Vn + 1) with the recommended value of 0.15 being used as the cut-off for selecting the suitable number of reference genes for qRT-PCR data normalization. A Vn/Vn + 1 higher than the threshold of 0.15 means that additional (n + 1) reference genes are necessary to normalize the genes.

**Figure 5:**
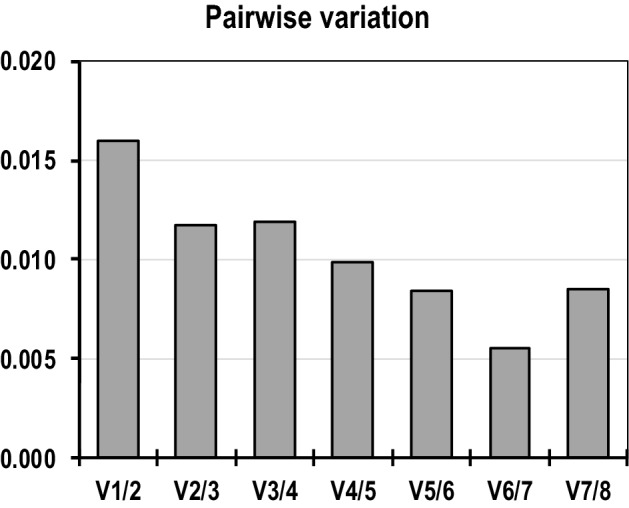
Optimal number of reference genes for normalization in antler blood samples of both young and adult red deer. A pair-wise variation (Vn/n + 1) analysis was used to determine the optimal number of reference genes required for accurate normalization. A value < 0.15 indicates that the use of additional reference genes would not markedly improve normalization.

As shown in Fig. 5, all pairwise variation values fell below a cut-off value of 0.15 in our study, indicating that the use of two reference genes would be sufficient. However, because all of the different algorithms revealed that B2M, ACTB and RPLP0 were the most stable genes, we selected all three as reference genes for normalization of our qRT-PCR data to increase the resolution and accuracy of results^37,47^ because we could not exclude the possibility that these stable genes might participate in other biochemical pathways other than antler development.

### Validation of reference genes

The analysis of the relative expression of three genes related to antler growth, ANXA2 (annexin 2), APOD (apolipoprotein D) and TPM1 (α-tropomyosin)^15,30,42^, was used to validate the stability of the best-ranked candidate reference genes (Supplementary Table S5 and Fig. 4).

To study the changes in the transcriptional expression profile of ANXA2, APOD and TPM1 in male red deer antler blood with ageing, we compared a group of five deer up to 4 years old (young group, our control group) with a group of ten older deer (adult group) by quantifying their mRNA levels and testing for statistical significance of differences in mRNA expression results in real-time qRT-PCR. First, the C_T_ for the target genes and for the three internal reference genes (B2M, ACTB and RPLP0) were determined for each sample in triplicate and the C_T_ averages calculated.

To normalize the differences in the amount of total cDNA added to each reaction, the efficiency of the retro-transcription step and the amplification efficiency for each primer pair, the differences in the C_T_ values obtained in the young and adult groups for the target gene (T) and the reference gene (R), denoted as ΔC_T(T)_ and ΔC_T(R)_, were estimated.

The variation factor was then assessed from the equation FV = (1 + E_T_)^ΔCT(T)^/(1 + E_R_)^ΔCT(R)^, where E_T_ and E_R_ are the PCR amplification efficiency of the target and the reference gene, respectively. The ΔCt for each experimental sample was subtracted from the ΔCt of the calibrator. Thus, all experimental samples are reported as relative transcription of the n-fold difference between the calibrator (young sample) and adult samples.

**Figure 6:**
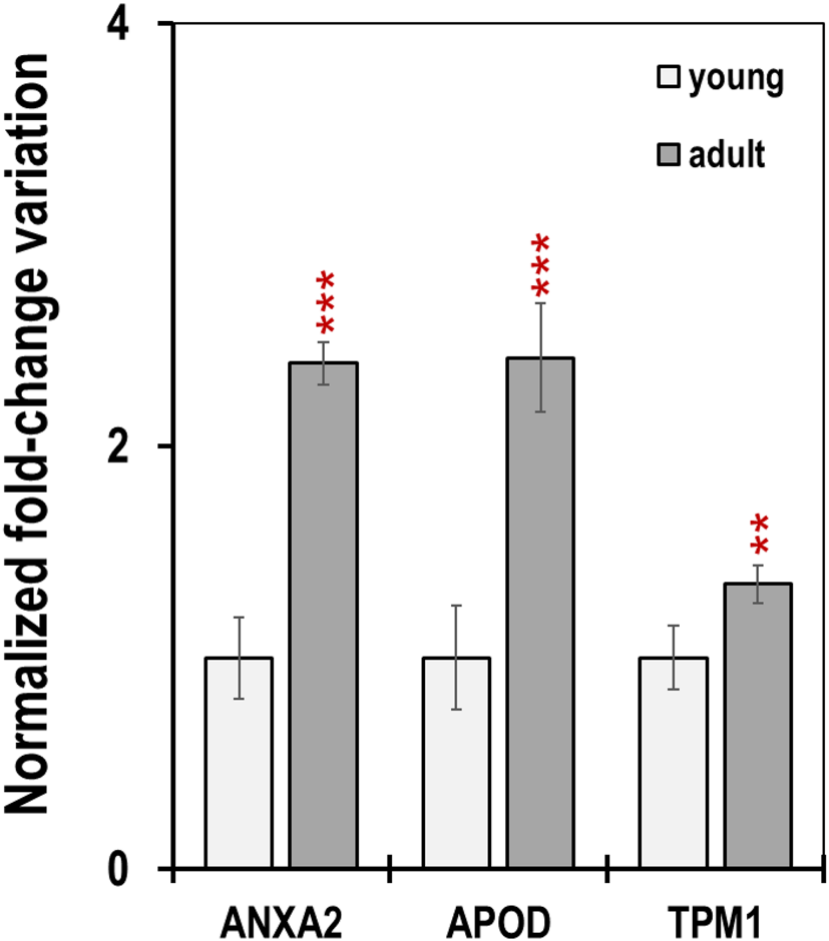
Normalized fold change variation in the expression level of ANXA2, APOD and TPM1 in the antler blood (mean ± SEM, n = 5 and n = 10 in the young and adult groups, respectively). The relative mRNA levels of three target genes, normalized with the geometric means of the abundance of the most stable reference genes B2M, ACTB and RPLP0. Statistical significance is expressed as: *p < 0.05, **p < 0.01 vs. control (young group).

Data shown in Fig. 6 indicate higher expression at the transcriptional level of the three analyzed genes in the adult antler blood.

Antler development is highly dependent on genetics and environmental conditions, but also on the age of the individual^3^. Age determines the beginning of annual antler casting and regeneration, with an average onset date for adult deer of almost 50 days earlier than for young individuals, leading to bigger antlers associated with a higher growth rate and a higher breeding success^3^.

Data in Fig. 6 show a > two-fold increased expression of ANXA2 and APOD and a < 30% increase in the expression of TPM1 in adults referred to young individuals.

ANXA2 expression has been associated with cell proliferation and plays an important role in regulating rapid growth and development^48^ of deer antler in the growing period^38^, as it is involved in the formation of calcium channels and in mineralization around hypertrophic chondrocytes and osteoblasts^49,50^.

APOD is a multifunctional and multi ligand binding protein, expressed in a wide variety of tissues. Its expression is increased after osteogenic differentiation and its deficiency is associated with high bone turnover, low bone mass, and impaired osteoblastic function in aged female mice^51,52^.

Finally, TPM1 was found to be a highly potent regulator of cell differentiation in tissue regeneration and immunomodulation^53^.

An enlarged expression of ANXA2, APOD, and TPM1 has been linked to the robust development of the antler in red deer^18^, but determinations were made in reserve mesenchyme, pre-cartilage and cartilage of the tip of the growing antlers. We demonstrate here that expression patterns of these genes in antler blood are a striking feature of the growing antler. Since they are involved in the robust bone development, the analysis of ANXA2, APOD and TPM1 might be indicative of an individual breeding success, and hence for potential strong sexual selection.

### The role of GAPDH, GUSB, PGK1 and SDHA in antler growth

**Figure 7:**
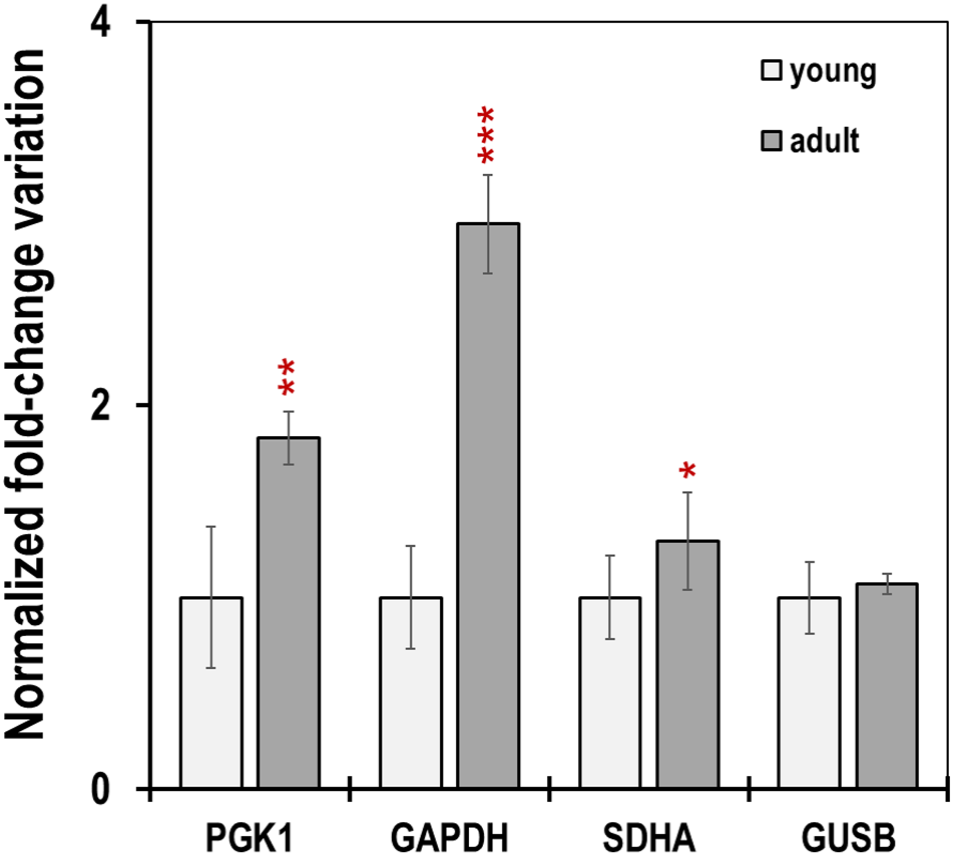
Normalized fold change variation in the expression level of PGK1, GAPDH, SDHA and GUSB in the antler blood (mean ± SEM, n = 5 and n = 10 in the young and adult groups, respectively).

The comprehensive statistical analysis of expression stability carried out in this work rejected GAPDH, GUSB, PGK1 and SDHA as reference genes to study antler growth in dehorned blood (Fig. 4, Supplementary Table S5). In other words, the expression of these four genes somehow contributes to the growth of the antler. Data in Fig. 7 indicate a significantly higher abundance of PGK1 and GAPDH and slightly increased levels of SDHA transcripts in adults.

Phosphoglycerate kinase 1 (PGK1) and Glyceraldehyde-3-phosphate dehydrogenase (GAPDH) are glycolytic enzyme involved in several metabolic pathways that are essential for cell growth and proliferation. The expression of these genes has been shown to differ in different tissue types and environment conditions^54,55^ because of its functions in transcriptional and posttranscriptional gene regulation, intracellular membrane trafficking, DNA replication and reparation^56,57^.

Succinate dehydrogenase (SDH) participates in both the tricarboxylic acid cycle and in the mitochondrial respiratory chain^58^.

The increased levels of transcripts of these genes indicated that antler growth in adults entails an increase of many metabolic pathways to adapt to the high energy demand during proliferation and to the specific cellular functions during bone growth^59^. We demonstrate here that antler blood is suitable for the determination of the expression patterns of these genes involved in robust bone development, which can be useful in research and farming strategies.

## Conclusions

In this study, we designed, optimized, and validated a two-step, real-time qRT-PCR protocol for the quantification of red deer mRNA abundance in antler blood (dehorned blood), following the MIQE Guidelines. Assay optimization included blood sample collection, pure and undegraded RNA obtention, total RNA retro-transcription and specific and accurate mRNA quantification by optimizing primer design, PCR assay conditions and identification of adequate genes for data normalization. To this respect, we concluded that qRT-PCR normalization to three stable genes (B2M, ACTB and RPLP0) is strongly convenient in experiments performed in the red deer antler blood. To our knowledge, this study is the first report on systematically evaluating the expression stability of different potential reference genes for qRT-PCR in red deer dehorned blood.

The validation of this proposal in dehorned blood showed that antler development is sustained by increased expression of genes linked to cell growth and proliferation (ANXA2, APOD and TPM1) and other linked to increased functioning of glycolysis and mitochondrial respiration (PGK1, GAPDH and SDHA) to generate the energy that fast and strong bone antler development needs. Our results point to dehorned blood as an excellent biological material to study the molecular mechanisms of antler development.

## Supplementary Information

Supplementary Information.

## Data Availability

All relevant data are within the paper and its Supporting Information files.
