# Supplementary material for: Evaluation of candidate reference genes for quantitative real-time PCR normalization in blood from red deer developing antlers
Source: Sci Rep. 2022 Sep 28;12:16264. doi: 10.1038/s41598-022-20676-9 (PMC9519901; doi:10.1038/s41598-022-20676-9)
Supplement: Supplementary file 1 — Supplementary Information. [file 41598_2022_20676_MOESM1_ESM.docx]

## **S****upplementary Table S1. Red deer specimens used for gene expression studies.**

| **Sample denomination** | **Origin** | **Age** | **Antler size (number of tips)** | **Experimental group** |
| --- | --- | --- | --- | --- |
| Y01 | Lagunes deer farm | 13-14 months | - | Young |
| Y02 | Lagunes deer farm | 13-14 months | - |  |
| Y03 | Cabañeros National Park | 3 years | 12 |  |
| Y04 | Cabañeros National Park | 3 years | 12 |  |
| Y05 | Cabañeros National Park | < 4 years | 15 |  |
| A01 | El Pardo Regional Park | > 4 years | 17 | Adult |
| A02 | El Pardo Regional Park | > 4 years | 15 |  |
| A03 | El Pardo Regional Park | > 4 years | 15 |  |
| A04 | El Pardo Regional Park | > 4 years | 16 |  |
| A05 | El Pardo Regional Park | > 4 years | 17 |  |
| A06 | El Pardo Regional Park | > 4 years | 19 |  |
| A07 | Cabañeros National Park | 5 years | 18 |  |
| A08 | Cabañeros National Park | 5 years | 20 |  |
| A09 | Cabañeros National Park | 6 years | 26 |  |
| A10 | Cabañeros National Park | 6 years | 20 |  |

## **Supplementary Table S2. Candidate reference genes information.**

| **Gene name** | **Gene symbol** | **Biological function** | **Ref.** |
| --- | --- | --- | --- |
| Succinate Dehydrogenase | SDHA | Oxidation of succinate in Krebs cycle and respiratory chain | [28] |
| Phosphoglycerate Kinase 1 | PGK1 | ATP-generating enzymes in glycolysis | [25] |
| Glyceraldehyde-3-Phosphate Dehydrogenase | GAPDH | Oxidoreductase in glucose metabolism | [26] |
| β-Actin | ACTB | Cytoskeletal structural protein | [18] |
| Ribosomal Protein Lateral Stalk Subunit P0 | RPLP0 | Protein biosynthesis | [28] |
| Glucuronidase Beta | GUSB | Degradation of glucuronic acid containing glycosaminoglycan | [28] |
| Beta-2-microglobulin | B2M | Component of the class I major histocompatibility complex (MHC). Involved in the presentation of peptide antigens to the immune system | [25] |
| Glucose-6-Phosphate 1-Dehydrogenase | G6PD | Catalyzes the rate-limiting step of the oxidative pentose-phosphate pathway, which represents a route for the dissimilation of carbohydrates besides glycolysis | [28] |

## **Supplementary Figure S1. Uncropped agarose gel (2%) electrophoresis depicted in Fig 2; the box points to the piece corresponding to Fig. 2a.**


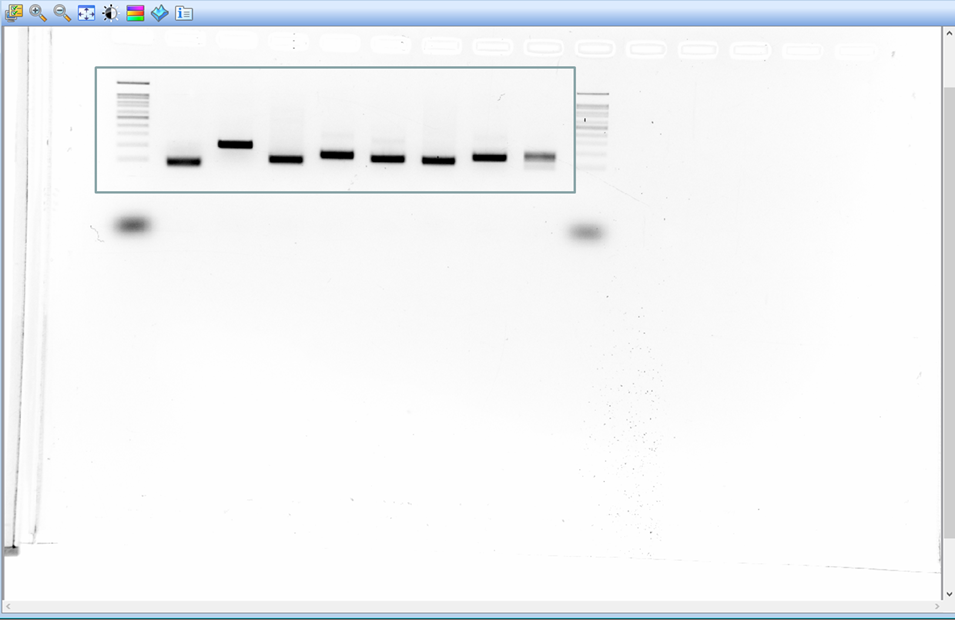


## **Supplementary Table S3. C_T_ values obtained for all samples and candidate reference genes.**

| **Sample** | **C_T_ values** | | | | | | | |
| --- | --- | --- | --- | --- | --- | --- | --- | --- |
|  | **B2M** | **ACT** | **RPLP0** | **G6PD** | **SDHA** | **PGK1** | **GUSB** | **GAPDH** |
| **Y01-1** | 14.47 | 16.40 | 15.28 | 27.53 | 25.26 | 19.15 | 23.56 | 17.13 |
| **Y01-2** | 14.29 | 16.38 | 15.34 | 27.52 | 24.33 | 19.07 | 23.62 | 16.99 |
| **Y01-3** | 14.42 | 16.47 | 15.31 | 27.53 | 24.80 | 19.11 | 23.38 | 17.06 |
| **Y02-1** | 14.63 | 17.13 | 16.12 | 28.63 | 25.19 | 19.70 | 24.13 | 18.17 |
| **Y02-2** | 14.46 | 17.02 | 16.02 | 28.83 | 25.64 | 19.77 | 24.14 | 18.07 |
| **Y02-3** | 14.52 | 17.19 | 16.11 | 28.73 | 25.42 | 19.74 | 24.13 | 18.12 |
| **Y03-1** | 14.98 | 17.06 | 16.00 | 27.81 | 23.44 | 19.80 | 22.98 | 18.29 |
| **Y03-2** | 15.12 | 17.08 | 16.08 | 27.62 | 23.36 | 19.80 | 23.11 | 18.55 |
| **Y03-3** | 15.08 | 17.18 | 16.12 | 27.72 | 23.40 | 19.80 | 23.05 | 18.42 |
| **Y04-1** | 16.01 | 18.21 | 16.77 | 28.67 | 23.83 | 20.50 | 23.58 | 19.27 |
| **Y04-2** | 16.01 | 18.11 | 16.62 | 28.30 | 24.22 | 20.50 | 23.53 | 19.53 |
| **Y04-3** | 16.02 | 18.22 | 16.70 | 28.49 | 24.03 | 20.50 | 23.85 | 19.40 |
| **Y05-1** | 14.38 | 16.72 | 15.28 | 28.14 | 23.99 | 19.42 | 23.14 | 17.71 |
| **Y05-2** | 14.17 | 16.65 | 15.27 | 28.17 | 24.30 | 19.28 | 23.06 | 17.48 |
| **Y05-3** | 14.40 | 16.43 | 15.28 | 28.16 | 24.15 | 19.35 | 23.13 | 17.60 |
| **A01-1** | 14.21 | 16.21 | 15.52 | 28.15 | 24.23 | 19.14 | 23.52 | 17.31 |
| **A01-2** | 14.27 | 16.03 | 15.80 | 28.06 | 24.11 | 19.32 | 23.38 | 17.32 |
| **A01-3** | 14.26 | 16.14 | 15.86 | 28.11 | 24.17 | 19.23 | 23.45 | 17.32 |
| **A02-1** | 14.18 | 15.93 | 15.51 | 27.92 | 23.67 | 18.24 | 23.22 | 15.79 |
| **A02-2** | 14.02 | 15.96 | 15.25 | 27.95 | 24.04 | 18.39 | 23.16 | 16.18 |
| **A02-3** | 13.94 | 15.84 | 15.54 | 27.94 | 23.86 | 18.32 | 23.19 | 15.99 |
| **A03-1** | 14.41 | 16.50 | 15.47 | 27.76 | 23.88 | 18.86 | 23.20 | 16.91 |
| **A03-2** | 14.23 | 16.55 | 15.39 | 27.64 | 23.87 | 19.01 | 23.18 | 16.79 |
| **A03-3** | 14.28 | 16.71 | 15.43 | 27.70 | 23.88 | 18.94 | 23.32 | 16.85 |
| **A04-1** | 14.72 | 17.06 | 16.26 | 27.93 | 23.91 | 19.02 | 24.23 | 16.34 |
| **A04-2** | 14.43 | 16.97 | 16.22 | 28.06 | 24.18 | 18.98 | 23.33 | 16.31 |
| **A04-3** | 14.44 | 16.77 | 16.24 | 28.00 | 24.05 | 19.00 | 23.27 | 16.33 |
| **A05-1** | 15.12 | 16.50 | 15.51 | 30.03 | 25.74 | 22.36 | 26.56 | 18.09 |
| **A05-2** | 15.10 | 16.55 | 15.25 | 30.38 | 25.93 | 22.31 | 26.39 | 18.09 |
| **A05-3** | 15.11 | 16.71 | 15.54 | 30.21 | 25.84 | 22.34 | 26.48 | 18.09 |
| **A06-1** | 14.19 | 15.86 | 15.91 | 28.01 | 24.08 | 18.44 | 24.19 | 15.63 |
| **A06-2** | 14.32 | 15.86 | 15.97 | 28.43 | 24.35 | 18.40 | 24.13 | 15.51 |
| **A06-3** | 14.32 | 15.98 | 16.02 | 28.22 | 24.22 | 18.42 | 24.16 | 15.57 |
| **A07-1** | 14.57 | 16.58 | 15.46 | 28.29 | 24.05 | 19.45 | 23.12 | 18.07 |
| **A07-2** | 14.56 | 16.98 | 15.47 | 28.55 | 23.74 | 19.55 | 22.98 | 18.02 |
| **A07-3** | 14.63 | 16.74 | 15.46 | 28.42 | 23.90 | 19.50 | 23.19 | 18.05 |
| **A08-1** | 14.86 | 17.13 | 16.06 | 27.90 | 23.44 | 19.51 | 23.56 | 18.20 |
| **A08-2** | 14.79 | 16.54 | 15.59 | 27.63 | 23.63 | 19.76 | 23.46 | 18.02 |
| **A08-3** | 14.64 | 16.41 | 15.83 | 27.77 | 23.54 | 19.64 | 23.50 | 18.11 |
| **A09-1** | 15.07 | 16.46 | 15.87 | 27.24 | 24.21 | 19.81 | 23.66 | 18.14 |
| **A09-2** | 15.09 | 16.37 | 15.84 | 27.17 | 24.06 | 19.72 | 23.70 | 18.12 |
| **A09-3** | 15.03 | 16.31 | 15.85 | 27.21 | 24.14 | 19.77 | 23.68 | 18.13 |
| **A10-1** | 14.33 | 16.31 | 15.04 | 27.64 | 25.02 | 19.91 | 23.46 | 17.98 |
| **A10-2** | 14.29 | 16.24 | 15.13 | 28.04 | 24.53 | 19.83 | 23.42 | 17.94 |
| **A10-3** | 14.35 | 16.36 | 15.02 | 27.84 | 24.78 | 19.87 | 23.44 | 17.96 |

## **Supplementary Table S4. Comparison of candidate reference gene expression stabilities by descriptive statistics using the XLSTAT v. 2020.2.2 software (Addinsoft) to calculate the statistical parameters of C_T_ values. Data in this table were used to obtain the boxplots shown in Fig. 3.**

| **Statistical parameter** | **Candidate reference gene** | | | | | | | |
| --- | --- | --- | --- | --- | --- | --- | --- | --- |
|  | **B2M** | **ACT** | **G6PD** | **GAPDH** | **GUSB** | **PGK1** | **RPLP0** | **SDH** |
| **No. of observations** | 45 | 45 | 45 | 45 | 45 | 45 | 45 | 45 |
| **Minimum value** | 13.940 | 15.840 | 27.170 | 15.510 | 22.980 | 18.240 | 15.020 | 23.360 |
| **Maximum value** | 16.020 | 18.220 | 30.380 | 19.530 | 26.560 | 22.360 | 16.770 | 25.930 |
| **Rank** | 2.080 | 2.380 | 3.210 | 4.020 | 3.580 | 4.120 | 1.750 | 2.570 |
| **1^st^ Quartile** | 14.290 | 16.310 | 27.720 | 16.910 | 23.190 | 19.020 | 15.390 | 23.880 |
| **Median** | 14.460 | 16.540 | 28.010 | 17.960 | 23.460 | 19.500 | 15.590 | 24.110 |
| **3^rd^ Quartile** | 14.980 | 16.980 | 28.300 | 18.120 | 23.700 | 19.800 | 16.020 | 24.350 |
| **Mean** | 14.638 | 16.640 | 28.134 | 17.532 | 23.687 | 19.567 | 15.725 | 24.276 |
| **Variance (n-1)** | 0.245 | 0.321 | 0.469 | 0.994 | 0.694 | 0.870 | 0.190 | 0.447 |
| **Standard deviation (n-1)** | 0.495 | 0.566 | 0.685 | 0.997 | 0.833 | 0.933 | 0.436 | 0.669 |
| **Coefficient of variation (n-1)** | 0.034 | 0.034 | 0.024 | 0.057 | 0.035 | 0.048 | 0.028 | 0.028 |
| **Skewness (Pearson)** | 1.356 | 1.116 | 1.726 | -0.366 | 2.503 | 1.531 | 0.503 | 1.056 |
| **Skewness (Fisher)** | 1.404 | 1.154 | 1.786 | -0.378 | 2.590 | 1.585 | 0.520 | 1.093 |
| **Kurtosis (Pearson)** | 1.571 | 1.418 | 3.390 | -0.388 | 5.889 | 2.905 | -0.398 | 0.312 |
| **Kurtosis (Fisher)** | 1.907 | 1.736 | 3.946 | -0.288 | 6.746 | 3.402 | -0.300 | 0.496 |
| **Standard error of the mean** | 0.074 | 0.084 | 0.102 | 0.149 | 0.124 | 0.139 | 0.065 | 0.100 |
| **Lower limit of the mean (95%)** | 14.490 | 16.470 | 27.929 | 17.233 | 23.437 | 19.287 | 15.594 | 24.075 |
| **Upper limit of the mean (95%)** | 14.787 | 16.810 | 28.340 | 17.832 | 23.937 | 19.848 | 15.856 | 24.477 |
| **Standard error of variance** | 0.052 | 0.068 | 0.100 | 0.212 | 0.148 | 0.186 | 0.040 | 0.095 |
| **Lower limit of variance (95%)** | 0.168 | 0.220 | 0.321 | 0.681 | 0.476 | 0.597 | 0.130 | 0.306 |
| **Upper limit of variance (95%)** | 0.391 | 0.511 | 0.748 | 1.586 | 1.108 | 1.389 | 0.303 | 0.714 |
| **Mean absolute deviation** | 0.379 | 0.421 | 0.461 | 0.813 | 0.526 | 0.626 | 0.370 | 0.501 |
| **Median absolute deviation** | 0.200 | 0.300 | 0.290 | 0.480 | 0.270 | 0.360 | 0.310 | 0.240 |
| **Geometric mean** | 14.630 | 16.631 | 28.127 | 17.504 | 23.674 | 19.547 | 15.719 | 24.267 |
| **Geometric standard deviation** | 1.034 | 1.034 | 1.024 | 1.059 | 1.034 | 1.047 | 1.028 | 1.028 |
| **Harmonic mean** | 14.623 | 16.622 | 28.119 | 17.475 | 23.661 | 19.527 | 15.713 | 24.258 |

## **Supplementary Table S5. Stability ranking of candidate reference genes.**

| **GeNorm** | | **NormFinder** | | **BestKeeper** | | **ΔCt Method** | | **RefFinder** | |
| --- | --- | --- | --- | --- | --- | --- | --- | --- | --- |
| ***Gene***  ***name*** | ***Stability value*** | ***Gene***  ***name*** | ***Stability***  ***value*** | ***Gene***  ***name*** | ***Standard deviation*** | ***Gene***  ***name*** | ***Average SD*** | ***Gene***  ***name*** | ***Geomean of ranking values*** |
| **B2M** | 0.354 | **B2M** | 0.314 | **RPLP0** | 0.370 | **B2M** | 0.66 | **B2M** | 1.19 |
| **ACTB** | 0.354 | **G6PD** | 0.484 | **B2M** | 0.379 | **ACTB** | 0.73 | **ACTB** | 2.06 |
| **RPLP0** | 0.398 | **ACTB** | 0.485 | **ACTB** | 0.421 | **G6PD** | 0.74 | **RPLP0** | 2.78 |
| **G6PD** | 0.587 | **PGK1** | 0.539 | **G6PD** | 0.461 | **RPLP0** | 0.78 | **G6PD** | 3.13 |
| **SDHA** | 0.672 | **RPLP0** | 0.573 | **SDH** | 0.501 | **PGK1** | 0.78 | **SDH** | 5.48 |
| **GUSB** | 0.705 | **SDH** | 0.612 | **GUSB** | 0.526 | **SDH** | 0.82 | **PGK1** | 5.60 |
| **PGK1** | 0.728 | **GUSB** | 0.648 | **PGK1** | 0.626 | **GUSB** | 0.82 | **GUSB** | 6.48 |
| **GAPDH** | 0.784 | **GAPDH** | 0.814 | **GAPDH** | 0.813 | **GAPDH** | 0.95 | **GAPDH** | 8.00 |
